# The Role of ATG16 in Autophagy and The Ubiquitin Proteasome System

**DOI:** 10.3390/cells8010002

**Published:** 2018-12-20

**Authors:** Qiuhong Xiong, Wenjing Li, Ping Li, Min Yang, Changxin Wu, Ludwig Eichinger

**Affiliations:** 1 Institute of Biomedical Sciences, Shanxi University, No.92 Wucheng Road, Taiyuan 030006, China; qxiong@sxu.edu.cn (Q.X.); liwenjing0607@163.com (W.L.); pingli@sxu.edu.cn (P.L.); minyang@163.com (M.Y.); 2 Center for Biochemistry, Medical Faculty, University of Cologne, Joseph-Stelzmann-Str. 52, 50931 Cologne, Germany

**Keywords:** ATG16, autophagy, ubiquitin proteasome system, UPS, crosstalk

## Abstract

Autophagy and the ubiquitin proteasome system (UPS) are the two major cellular degradation pathways, which are critical for the maintenance of cell homeostasis. The two pathways differ in their mechanisms and clients. The evolutionary conserved ATG16 plays a key role in autophagy and appears to link autophagy with the UPS. Here, we review the role of ATG16 in different species. We summarize the current knowledge of its functions in autophagosome membrane expansion and autophagosome formation, in Crohn’s disease, and in bacterial sequestration. In addition, we provide information on its autophagy-independent functions and its role in the crosstalk between autophagy and the UPS.

## 1. Introduction

Cell homeostasis is maintained by a precisely regulated balance between synthesis and degradation of cellular components. Macroautophagy (hereafter referred to as autophagy for simplicity), chaperone-mediated autophagy (CMA), and the ubiquitin proteasome system (UPS) are the major routes for protein and organelle clearance in eukaryotic cells. In CMA, an important pathway in cell proteostasis, cytosolic proteins bearing a pentapeptide motif are directly delivered to the lysosome for degradation [1]. The UPS is responsible for the degradation of many regulated, short-lived, abnormal, denatured, or, in general, damaged proteins [2]. However, the proteasome does not have the ability to degrade aggregated proteins. It has been found that protein aggregates are cleared by autophagy and this process is called aggrephagy. Furthermore, cellular organelles, invading microorganisms as well as most of the long-lived proteins are degraded via autophagy [3].

In the 1990s, genetic studies in yeast identified a series of autophagy-related (ATG) genes. Now the number of ATG genes has increased to more than 37 [4]. Among them, 15 ATG genes have been referred to as “core” ATG genes, as they are required for autophagosome formation [5]. They can be subdivided in four subgroups [5,6]: (1) the ATG1/ULK1 complex, composed of ATG1/ULK1, ATG13, ATG101 and FIP200 (focal adhesion kinase family interacting protein of 200 kDa). FIP200 increases the ATG1/ULK1 kinase activity and stability [7]; (2) the class III PI3K (phosphatidylinositol 3-kinase) complex composed of Vps34, Vps15, ATG6/Beclin1 and ATG14. Both complexes regulate the initiation phase of autophagy; (3) the two Ubl (ubiquitin-like) protein conjugation systems which consist of ATG12, ATG5, ATG16, ATG8/LC3, ATG7, ATG10, ATG3, and ATG4 and are important for autophagosome formation and membrane elongation; and (4) two transmembrane proteins, ATG9 (and associated proteins ATG2 and ATG18) and VMP1 which are important for autophagosome formation and maturation [8]. ATG16 is one of the core autophagy proteins, it associates non-covalently with the ATG12~5 conjugate and forms a hetero-tetrameric complex [9,10]. In this complex, the ATG12~5 conjugate holds the E3-ligase activity to promote the covalent linkage of ATG8/LC3 to the lipid phosphatidyl-ethanolamine (PE). For this activity ATG16 is not strictly required [11]. ATG16 is present in all eukaryotic species and its function is well-studied. ATG16 is required for the correct localization of the ATG12~5 conjugate to the pre-autophagosomal structure [12] in yeast and to the isolation membrane in higher eukaryotes [13,14]. In ATG16L1-deficient mouse embryonic fibroblast (MEFs), the ATG12~5 conjugate could not be recruited to the isolation membrane, which resulted in a loss of ATG8/LC3 lipidation [15]. It is thought that the ATG16 complex may deliver ATG8/LC3 to the forming autophagosome and subsequently ATG8/LC3 functions in the closing of the autophagosome [16]. In this review, we will describe ATG16 in different models and focus on the functions of ATG16 and its role in connecting autophagy and the UPS.

## 2. ATG16 is Ubiquitous in Eukaryotes and Highly Conserved

**Figure 1:**
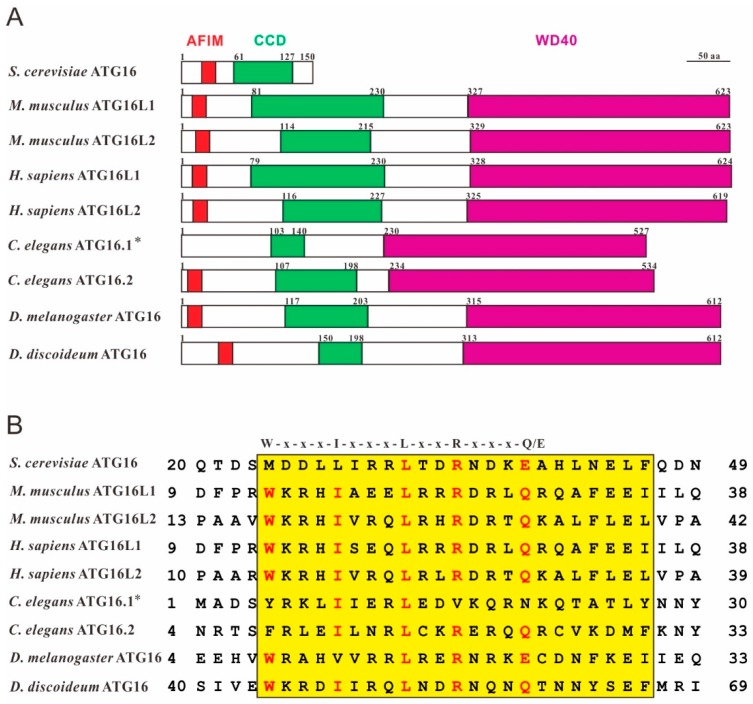
ATG16 shares high structural similarity in different species. (**A**) Domain structures of *Saccharomyces cerevisiae* ATG16 (NP_013882.1), *Homo sapiens* ATG16L1 (NP_001350671.1) and ATG16L2 (NP_203746.1), *Mus musculus* ATG16L1 (NP_001192320.1) and ATG16L2 (NP_001104581.1), *Caenorhabditis elegans* ATG16.1 (NP_508768.1) and ATG16.2 (NP_495299.2), *Drosophila melanogaster* ATG16 (NP_001138124.2) and *Dictyostelium discoideum* ATG16 (XP_643673.1). Conserved domains were predicted using SMART (http://smart.embl-heidelberg.de/). AFIM (ATG5-interacting motif), red; CCD (coiled-coil domain), green; WD40 (tryptophan-aspartic acid, WD40, repeat domain), violet. * the sequence used here lacks the N-terminal 51 amino acids because it was shown that the *C. elegans atg-16.1* gene encodes 527 amino acids and not, as originally predicted 578 amino acids [18]. (**B**) Sequence alignment of the AFIM regions of different ATG16 proteins using the Multiple Sequence Alignment program at the NCBI (https://blast.ncbi.nlm.nih.gov/Blast.cgi). The highly conserved AFIM residues are denoted on the top [19]. Red letters highlight conserved key residues of the AFIM and yellow shading indicates the highly conserved AFIM region between species.

All eukaryotic species that were analyzed harbor a gene for ATG16 and the encoded protein shows high structural and sequence similarity across species (Figure 1). Most lower eukaryotes only have a single gene for ATG16 while most higher eukaryotes encode two ATG16 paralogs. The two paralogs seem to have independent functions, although they share similar structures and even form a complex with the ATG12~5 conjugate [17].

### 2.1. ATG16 in Yeast

ATG16 (Apg16p) was first identified in yeast in 1999 by the group of Ohsumi [10]. Yeast ATG16 is a 150 amino acid protein that contains an N-terminal AFIM (ATG5-interacting motif) and a C-terminal CCD (coiled-coil domain) but lacks the tryptophan-aspartic acid (WD40) repeat domain that constitutes the C-terminal domain in ATG16 proteins of most species. It association via the AFIM with ATG5 and its CCD, which mediates the formation of the ATG12~5/16 heterotetrameric complex, is important for autophagy in yeast [9,10,20].

### 2.2. ATG16 in Mouse and Human

Mouse and human ATG16L1 and L2 are highly conserved and share 94 and 83% sequence identity, respectively. Mouse ATG16 was identified in 2003 by the same group as yeast ATG16 [21]. Mouse and human ATG16 shows homology to yeast ATG16 in its N-terminal region, but harbor in addition a large C-terminal domain with seven WD40 repeats, which is missing in the yeast protein. Therefore, they named the protein ATG16L (ATG16-like protein) or ATG16L1. Because the function of ATG16L1 is similar to yeast ATG16, it was concluded that mouse ATG16L1 and yeast ATG16 are orthologs [21]. In 2011, Ishibashi et al. identified a new isoform of ATG16L in mouse and named it ATG16L2. Similar to ATG16L1, ATG16L2 can interact with ATG5 through the AFIM and also self-oligomerizes via the CCD, but it is unable to mediate canonical autophagy [17]. 

Human ATG16L1 was identified by large-scale sequencing analysis of a human fetal brain cDNA library [22]. Database searching revealed that there exist at least four splice variants [22] and Jiang et al. suggested the presence of seven splice variants of ATG16L1 in *H. sapiens* [23]. Functional analysis of three isoforms revealed different autophagic properties because of the absence of some regions of ATG16L1 which impaired their localization on autophagosomes [23]. For ATG16L2 it was shown that mRNA and protein levels decreased in Multiple Sclerosis (MS) patients. The authors suggested that ATG16L2 may play an important role in autophagy of T cells and may serve as a potential biomarker for the prediction of relapse rates of MS patients [24].

### 2.3. ATG16 in C. Elegans

*C. elegans* ATG16 was identified by genetic screens in 2010 [25]. *C. elegans* has two ATG16 paralogs, ATG16.1 and ATG16.2 and both proteins have the same domain structure including the seven WD40 repeats at the C-terminus as human ATG16 (Figure 1A). Depletion of either *atg16.1* or *atg16.2* caused defects in autophagy, growth, and development. Interestingly, the phenotype was much more severe in the double mutants, suggesting that ATG16.1 and ATG16.2 have partially overlapping functions [18]. Furthermore, protein sequence alignment showed that four of the five critical amino acids of the AFIM of ATG16.2 were conserved, while only two of them were conserved in ATG16.1 (Figure 1B). Functional analysis, however, showed that the N-terminus of ATG16.1 still interacted with ATG5 [18]. In contrast to mammalian and yeast ATG16, neither ATG16.1 nor ATG16.2 appear to be required for LGG-1/ATG8 (LC3 in mammals) lipidation, but ATG16.2 is required for lipidated LGG-1/ATG8 to form punctate structures [18]. 

### 2.4. ATG16 in D. Discoideum

*Dictyostelium* ATG16 was identified in a screen for developmental mutants and the gene was originally named *tipD* because the encoded protein was required for tip formation during multicellular development [26]. The *tipD* gene encodes an ATG16 ortholog and later it turned out that the developmental phenotype observed in the *tipD^−^* mutant is typical for many *D. discoideum* autophagy mutants and similar developmental phenotypes were described for knockout mutants of different autophagy genes, such as e.g., *atg5*, *atg7* and *atg9* [8,27,28,29] Furthermore, we found that ATG16 knockout cells display a pleiotropic phenotype [30,31]. In contrast to yeast ATG16, *D. discoideum* ATG16 contains, as is the case for the orthologs of higher eukaryotes, seven WD40 repeats in the C-terminal half of the protein (Figure 1A).

## 3. Functions of ATG16

ATG16 is composed of three distinct regions—the N-terminal portion containing the AFIM, followed by the CCD and seven WD40 repeats in the C-terminal half. Each domain has its distinct binding partners which mediate specific functions. In particular, the WD40 domain, which folds into a β-propeller structure is known as a hub for protein-protein interactions [12,32,33]. Consequently, many proteins that interact with ATG16 have been identified so far in different screens (Table 1). These interactions appear to be crucial for autophagy-dependent or -independent functions of ATG16 and will be dealt with below.

### 3.1. ATG16 in Autophagosome Formation

**Figure 2:**
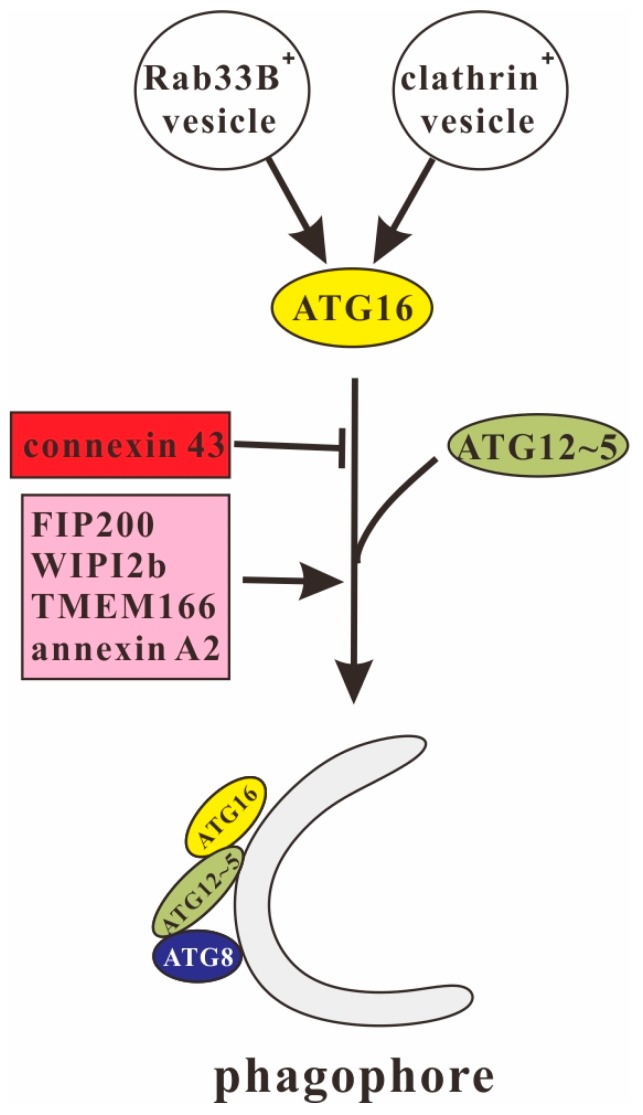
ATG16 in the initiation phase of autophagosome formation. The ATG12~5/16 complex is required for ATG8/LC3 lipidation and is recruited to the phagophore or isolation membrane by interacting with FIP200, WIPI2b and TMEM166. ATG16 also interacts with Rab33B, clathrin, annexin A2 and connexin 43. The former three promote autophagosome membrane expansion while the latter is inhibitory.

Formation of the ATG12~5/16 complex is promoted by phosphorylation on Ser139 [57] and inhibited by methylation on Lys151 in cardiomyoctyes [58]. Binding of the complex to the phagophore or omegasome appears to be mediated by different binding partners. The class III PI3K VPS34 is essential for autophagosome formation and it was shown that the yeast ATG16 complex binds to PI3P-containing liposomes [59]. Thus, the local production of PI3P on autophagosome precursors may contribute to the localization of the ATG16 complex on the phagophore assembly site (PAS) or isolation membrane [60]. However, the mechanism how the ATG16 complex is recruited to the PAS or isolation membrane is still not completely solved, because neither ATG5 nor ATG12 nor ATG16 show any typical membrane-binding motifs [61]. Recently, two independent studies identified FIP200 as a direct binding partner of ATG16L1 [34,35]. The FIP200-binding domain (FBD) of mammalian ATG16L1 has been mapped to the region between its CCD and WD40 repeat domain. It is thought that FIP200 is responsible for the specific recruitment of ATG16L1 complex to the isolation membrane [34]. In another study, WIPI2b, the mammalian homolog of *S. cerevisiae* ATG18, was also shown to bind to ATG16L1. Interestingly, in ATG16L1-deficient MEFs, the expression of an ATG16L1 mutant that was able to bind FIP200, but defective for binding WIPI2b, LC3 lipidation was not rescued. This suggests that WIPI2b rather than FIP200 binding to ATG16L1 is responsible for its recruitment to the isolation membrane and its function in autophagy [36]. In addition, the transmembrane protein 166 (TMEM166/EVA1A) was also shown to promote the recruitment of ATG12~5/16L1 complex to the autophagosome membrane and enhance the formation of the autophagosome (Figure 2) [37]. 

Rab33B is a Rab-type small GTPase which is ubiquitously expressed, localizes to the cis-Golgi and functions in intra-Golgi trafficking and retrograde Golgi-ER trafficking [62,63]. Itoh et al. showed that Rab33B can interact with the CCD of ATG16L1 in a GTP-dependent manner [38]. A GTPase-deficient mutant of Rab33B enhanced LC3 lipidation, even under nutrient-rich conditions. In addition, expression of the Rab33B-binding domain of ATG16L1 or silencing of Rab33B decreased ATG12- and ATG8/LC3-positive puncta, which suggested the inhibition of autophagosome formation [38,39]. It is, therefore, likely that ATG16L1 functions as a specific effector molecule for Rab33B in autophagosome formation (Figure 2) [40].

Clathrin is a major component of coated vesicles that mediate sorting and selective transport of membrane-bound proteins for several pathways of intracellular membrane traffic. Clathrin-coated vesicles (CCVs) are responsible for receptor-mediated endocytosis (RME) at the plasma membrane and sorting of proteins at the trans-Golgi network (TGN) during the biogenesis of lysosomes and secretory granules [64]. Ravikumar et al. showed that the heavy chain of clathrin interacts with the N-terminal region of ATG16L1 and is involved in the formation of ATG16L1-positive early autophagosome precursors [42]. Knock down of the clathrin heavy chain resulted in an inhibition of both autophagosome formation and maturation [41,42,43]. Furthermore, annexin A2 was also demonstrated to promote phagophore assembly by enhancing ATG16L1-positive vesicle formation and homotypic fusion [44,65,66]. In contrast, connexin 43 (Cx43), a main component of plasma membrane gap junctions, inhibited autophagosome formation through interaction with the WD40 repeat domain of ATG16L1 (Figure 2) [45,46].

### 3.2. ATG16 in Crohn’s Disease

Crohn’s disease [67] is a type of inflammatory bowel disease that causes diarrhea, malabsorption, fistulation, and intestinal obstruction [68]. The pathophysiology of CD is poorly understood. Recently, a coding polymorphism in the *atg16L1* gene that codes for a threonine to alanine switch (T300A) was identified as a risk factor for the development of CD in Caucasian people [69,70]. In contrast, a genome-wide association study of Crohn’s disease in the Korean population identified an association with *atg16L2* [71,72]. 

Paneth cells are specialized intestinal epithelial cells that secrete granules containing antimicrobial peptides such as α-defensins, secretory PLA2, and lysozyme [73]. In *Drosophila*, ATG16 is essential for the differentiation of intestinal stem cells into entero-endocrine (EE) cells. Expression of ATG16 lacking the WD40 repeat domain resulted in morphological changes in the intestine that resembled inflammatory bowel disease (IBD) [74]. Furthermore, ATG16L1 hypomorphic mice that expressed the ATG16L1^T300A^ mutation showed defects in the granule exocytosis pathway of the Paneth cell and revealed abnormal Paneth cell morphology [75]. It was also demonstrated that the expression level of the ATG16L1^T300A^ variant decreased and that it had an increased sensitivity to caspase-3 mediated cleavage [76,77]. Interestingly, the most common Crohn’s-associated NOD2 variant was defective in recruitment of ATG16L1 to the bacterial entry site and in bacterial autophagy [50,51]. The lower levels of ATG16L1 may lead to lower ATG16L1-NOD2 complex formation. This could shift the balance of NOD2 functional effects towards increased receptor-interacting protein 2 (RIP2) kinase activation, which would subsequently cause an up-regulation of IL-1β, IL-6 and IL-18 mRNA expression [15,77,78]. ATG16L1 may therefore modulate the balance between NOD2-induced xenophagy versus cytokine production. This may explain the effects of this polymorphism on the inflammatory process in CD [78].

### 3.3. ATG16 in Bacterial Sequestration

**Figure 3:**
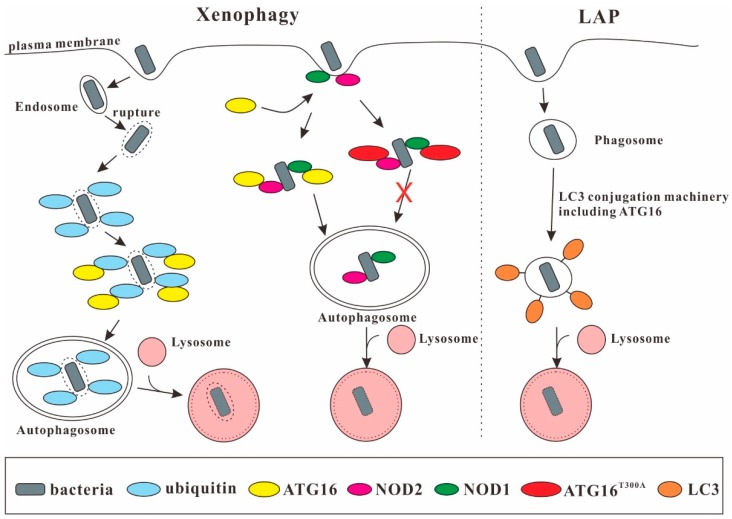
ATG16 in xenophagy and LC3-associated phagocytosis (LAP). Through interaction with ubiquitin, NOD1, and NOD2, ATG16 is important for xenophagy. The ATG16^T300A^ variant has a decreased stability and is impaired in this process. ATG16 is also required for LAP, which promotes LC3 lipidation of the phagosomal membrane.

ATG16 comprises seven C-terminal WD40 repeats that are dispensable for canonical autophagy [79,80] but are important in xenophagy. One recent study showed that the human transmembrane protein TMEM59 which localizes to the endosomal compartment can directly bind the WD40 repeat domain of ATG16L1 via a minimal 19 amino acids subdomain in its cytoplasmic domain [47,48]. The same motif has also been found in the cytoplasmic region of Toll-like receptor 2 (TLR2), which promotes ATG8/LC3 labeling of conventional phagosomes [81], in the N-terminal caspase recruitment domain of NOD2 and in two additional proteins, (T3JAM and DEDD2), not previously linked to autophagy [47]. Surprisingly, NOD1 lacks the motif but can also bind directly to the WD40 repeats of ATG16L1 [49]. NOD1 and NOD2 are involved in bacterial sensing through recognition of peptidoglycan and provide a link to autophagy by recruiting ATG16L1 to the entry site of *Shigella flexneri* at the plasma membrane [50]. Along this line ATG16L1 is required for the defense of intestinal epithelial cells against *Salmonella typhimurium* [82]. During infection with *S. typhimurium*, endosomal proteins, which are exposed through damage of the endosomal membrane by bacteria residing in the endosome, are ubiquitinated and autophagy is triggered. In this process, ATG16L1 is recruited to the *Salmonella*-containing endosomes by a direct interaction between the WD40 repeat domain and ubiquitin [52]. The T300A mutation had no effect on ATG16L1 binding to ATG5 or on basal autophagy. However, the T300A variant showed impaired xenophagy against *S. typhimurium* [83] and enhanced replication of adherent-invasive *Escherichia coli* (AIEC) (Figure 3) [84]. ATG16L1 is also required for LC3-associated phagocytosis (LAP) as murine bone marrow-derived macrophages deficient in ATG16L1 failed to undergo both, canonical autophagy or LAP [85,86].

Autophagy is generally considered to be antipathogenic. However, there are also reports that autophagy is exploited by the pathogens. One recent study demonstrated that the ATG16L1^T300A^ variant conferred protection from cellular invasion by *S. typhimuriunm* in HCT116 cells [87]. Wang et al. also demonstrated that Atg16L1 deficiency resulted in protection of these mice against both acute and latent uropathogenic *E. coli* (UPEC) infection [75,88,89]. Furthermore, for HeLa cells and MEFs infected with *Staphylococcus aureus* autophagy was important for the survival and replication of the pathogen [90]. In HeLa cells infected with *S. aureus*, TMEM59 promoted ATG8/LC3 conjugation to the single-membrane bacterial phagosome by directly engaging ATG16L1. TMEM59 depletion blocked ATG8/LC3 lipidation and resulted in reduced bacterial recovery from infected cells [47]. Thus, the role of ATG16L1 in microbial infection apparently depends on the type of invading microbe and the cell type [87].

### 3.4. ATG16 and the UPS

**Figure 4:**
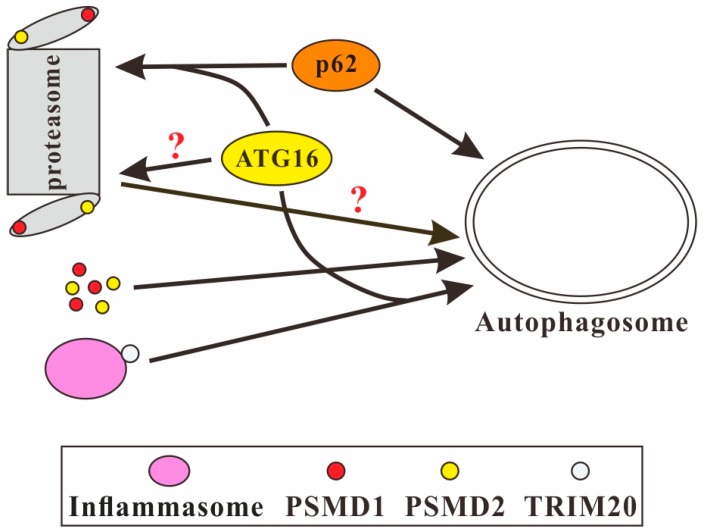
ATG16 links autophagy and the UPS. ATG16 facilitates the proteasomal degradation of p62/SQSTM1 and the autophagic degradation of the inflammasome via TRIM20. Furthermore, ATG16 mediates the autophagic degradation of the proteasomal subunits PSMD1 and PSMD2 in *D. discoideum* and might also mediate the autophagic degradation of the proteasome. Vice versa, ATG16 itself maybe a substrate of the UPS.

WD40 repeats are present in a wide range of proteins involved in distinct biological activities [32,33]. A large subfamily of WD40 repeats has been identified as distinct ubiquitin-binding domains that interact with ubiquitin in a similar fashion [12]. The WD40 repeat domain of ATG16 can bind to ubiquitin in vitro and in vivo [49,52]. ATG16 is also involved in Cullin-3-mediated ubiquitination and degradation of p62/SQSTM1 by the proteasome. p62/SQSTM1 acts as a selective autophagy adaptor for autophagic degradation of ubiquitinated protein aggregates. It also interacts with the proteasome and thus shuttles proteins for proteasomal degradation [91]. Here, ATG16 determined the neddylation of Cullin-3, which is an E3 ubiquitin ligase for p62/SQSTM1. In the absence of ATG16, endogenous Cullin-3 was not neddylated, which resulted in accumulation of p62/SQSTM1 (Figure 4) [92]. Furthermore, ATG16 itself is possibly a client of the UPS. The ATG16L1 protein level increased in *Atg5* knockout MEFs after treatment with proteasome inhibitors [80]. In addition, ATG16 is also crucial for optimal UPS function, since the *Dictyostelium atg16* knockout mutant displayed strongly reduced proteasomal activity [30].

### 3.5. Autophagy-Independent Roles of ATG16

**Figure 5:**
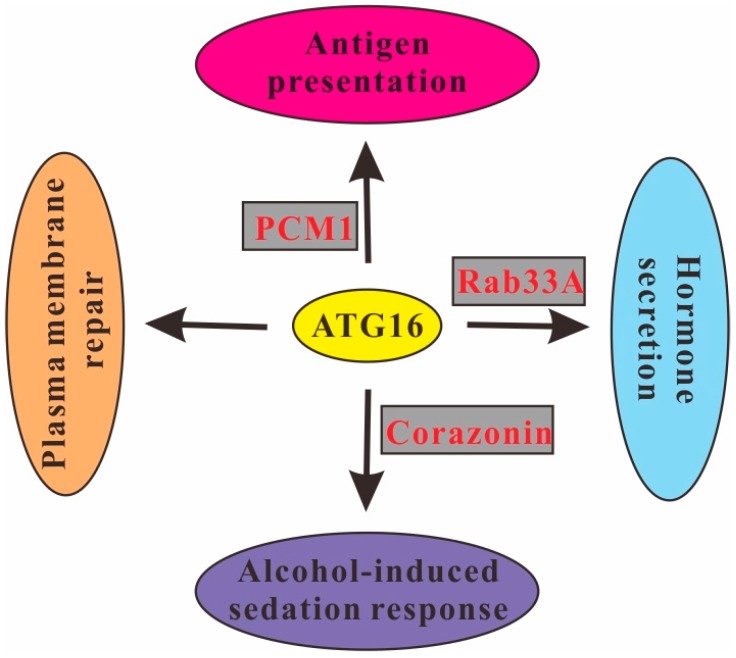
Autophagy-independent roles of ATG16. ATG16 is required for plasma membrane repair in MEFs and regulates antigen presentation in human B cells, hormone secretion in PC12 cells and the alcohol-induced sedation response in *Drosophila* through interaction with PCM1, Rab33A and activation of the expression of Corazonin, respectively.

The WD40 repeat domain is dispensable for canonical autophagy but is required for other processes [93]. Its deletion inhibited MHC class II antigen presentation in mouse dendritic cells during influenza A virus infection [79]. Recent studies further demonstrated that the interaction of ATG16L1 and the microtubule-organizing center-associated protein pericentriolar material 1 (PCM1) is required for B-cell receptor (BCR) polarization which is crucial for antigen presentation in the human B-cell line BJAB [53]. In addition, the coiled-coil domain of ATG16L1 also functions in hormone secretion in PC12 cells by binding to Rab33A. This interaction is required for the hormone-containing dense-core vesicle localization of ATG16L1, which is independent of its autophagic activity [54]. The *Drosophila* neuropeptide Corazonin is thought to be the homolog of mammalian gonadotropin-releasing hormone, the production of which may promote the sedation response during ethanol exposure. ATG16 expression in Corazonin-producing neurosecretory cells promoted ethanol sedation. In contrast, *Drosophila* deficient for ATG16 showed not only a defect in autophagy but also decreased expression of Corazonin and an increased resistance to the sedative effects of ethanol. This effect was not seen in flies deficient for ATG7 or ATG3, suggesting that ATG16 has an autophagy-independent function in the alcohol-induced sedation response in *Drosophila* [94]. Very recently, a study showed that ATG16 is required in MEFs for autophagy-independent plasma membrane repair [67] (Figure 5).

## 4. Crosstalk between Autophagy and the UPS

Autophagy and the UPS were thought to act independently for a long time, but evidence is accumulating that these two major intracellular pathways for protein and organelle clearance in eukaryotic cells are interrelated. It is well established that there is compensatory up-regulation of autophagy upon inhibition of proteasomal activity [95,96,97,98]. However, the effect of autophagy inhibition on the UPS is less clear. Wang et al. reported an up-regulation of proteasomal subunits and an increase in proteasomal activity in response to pharmacological inhibition of autophagy as well as downregulation of autophagy genes by RNAi in colon cancer cells [99]. In contrast, autophagy inhibition in HeLa cells resulted in impaired clearance of UPS clients although proteasomal activity was unchanged [100]. The authors suggest that autophagy inhibition leads to p62/SQSTM1 accumulation, which, in turn, impairs flux through the UPS. Of note, reduced proteasomal activity and an accumulation of ubiquitinated proteins have also been observed in case of autophagy inhibition in neuroblastoma cells and in autophagy-deficient *D. discoideum* cells [30,101,102]. Furthermore, proteasomal activity was also significantly reduced in mice deficient for the lysosomal enzyme cathepsin D [103].

Ubiquitination has been deciphered as the hallmark for proteins to be degraded by the 26S proteasome. Several recent studies have shown that ubiquitin also plays an essential role in selective autophagy by acting as a sorting tag for incorporation of autophagy substrates [104]. Thus, both systems, selective autophagy and the UPS, use ubiquitin as an important substrate recognition signal, and different ubiquitin codes appear to distinguish between selective autophagy and the UPS [105]. Lys48 (K48) polyubiquitin chains are the most common signal for proteasomal degradation, while monoubiquitination and/or oligomeric Lys63 (K63)-linked ubiquitin chains mark substrate proteins for degradation via autophagy [105,106]. However, the code is not unambiguous: the autophagic machinery can also target ubiquitin linkages besides K63- and linear-ubiquitin chains [52] and the K63-polyubiquitinated tau can be delivered by p62/SQSTM1 to the proteasome for degradation [107]. In addition, several proteins, as e.g., α-synuclein and other aggregate-prone proteins, are known to be substrates of both degradative pathways [108,109].

It was shown that ULK1/ATG1 and ATG8/LC3 can be degraded by the proteasome in an ubiquitin-dependent and -independent way [110,111]. Vice versa, proteasomal subunits were found to be degraded by lysosomes [112]. In contrast to proteasomal degradation, selective autophagy needs cargo adaptors, such as p62/SQSTM1, NBR1, ALFY, HDAC6, Parkin and NDP52, to mediate the interaction between ubiquitin and the autophagic machinery [3,113]. Recently, Marshall et al. demonstrated in *Arabidopsis thaliana* that inactive 26S proteasomes were ubiquitinated and then degraded via autophagy, a process the authors termed proteaphagy. In *A. thaliana* it is mediated by the proteasomal subunit RPN10/PSMD4 and ATG8/LC3. However, the interaction of RPN10/PSMD4 and ATG8/LC3 was not found in yeast and mammals, suggesting that in these organisms other adaptors or receptors mediate proteaphagy [114]. Recent studies showed that in HeLa cells proteaphagy was mediated by p62/SQSTM1 [115] and in yeast by the ubiquitin receptor Cue5 and sorting nexin 4 (Snx4) [116]. Furthermore, Waite et al. reported that degradation of the yeast proteasomal core (20S) and regulatory particle (19S) subunits were different upon nitrogen starvation. It appears that the 20S core particles are degraded by autophagy in an Ubp3-dependent and the 19S regulatory particles in an Ubp3-independent way, respectively [117].

## 5. ATG16 Mediates the Crosstalk between Autophagy and UPS in *Dictyostelium*

ATG16 contains three distinct domains, and each of them can bind to different partners and has specific functions. As a common protein interaction domain, the WD40 repeat domain binds to ubiquitin and several other proteins independent of ubiquitin. This likely extends the functions of ATG16 to also serve as an adaptor as p62/SQSTM1 does. It is also feasible that ATG16 directly recognizes autophagy substrates as a receptor to mediate degradation by autophagy. Kimura et al. showed that tripartite motif 20 (TRIM20) mediates the autophagic degradation of inflammasome components by interacting with the WD40 repeat domain of ATG16L1 (Figure 4) [55]. Recent studies in *D. discoideum* showed that the N-terminal half of ATG16 interacted with the 19S proteasomal subunit PSMD1 and the C-terminal half containing the WD40 repeat domain interacted with PSMD1 and PSMD2. Further analysis showed that RFP-PSMD1 and RFP-PSMD2 colocalized with the autophagosome marker GFP-ATG8/LC3 and with ATG16-GFP. In addition, RFP-PSMD1 and RFP-PSMD2 were delivered to lysosomes for degradation. These results indicated that ATG16 mediates the autophagic degradation of PSMD1 and PSMD2 in *D. discoideum* (Figure 4) [56]. Further work will unravel whether the 19S regulatory particle or the 26S proteasome are degraded via autophagy and whether ATG16 indeed acts as an adaptor or receptor for proteaphagy.

## 6. Final Remarks

Autophagy and UPS are crucial for protein and cell homeostasis. As a core autophagy protein, ATG16 plays a pivotal role in canonical autophagy processes, such as autophagosome formation. ATG16 is composed of three distinct domains—the N-terminal region which includes the AFIM, the CCD which mediates dimerization and the C-terminal WD40 repeat domain which is crucial for many protein-protein interactions. The N-terminal region and the CCD of ATG16 are indispensable for canonical autophagy. The C-terminal WD40 repeat domain seems to have additional functions, such as in bacterial sequestration and Crohn’s disease. Furthermore, ATG16 also has functions besides its role in canonical autophagy. It plays a role in macropinocytosis, in the uptake of the human pathogen *Legionella pneumophila* and in phagocytosis [30]. ATG16 also links autophagy and the UPS, as proteasomal activity is strongly decreased in ATG16 deficient cells and as it mediates the autophagic degradation of the proteasomal subunits PSMD1 and PSMD2 in *D. discoideum* [30,56]. It is expected that in the years to come more and more of the fascinating roles and interactions of ATG16 in canonical and non-canonical autophagy and in autophagy-independent processes will be unraveled.

## Figures and Tables

**Table 1:** ATG16 interacting proteins, the binding regions, and the functions of this interactions.

Interacting Protein	Binding Region of ATG16	Function	References
ATG5	N-terminal AFIM	Essential for ATG16 complex localization to PAS and ATG8/LC3 lipidation	[9,11,14,15]
FIP200	Between CCD and WD40 repeat domain	Promotes the specific targeting of ATG16L1 to the isolation membrane	[34,35]
WIPI2b	CCD	Promotes the recruitment of ATG16 to the isolation membrane	[36]
TMEM166	WD40 repeat domain	Promotes the recruitment of ATG16L1 complex to the autophagosome membrane	[37]
Rab33B	CCD	Transport of Golgi-derived membrane to autophagosome	[38,39,40]
Clathrin	N-terminal region	Transport of plasma membrane to autophagosome	[41,42,43]
Annexin A2	ND	Promotes the formation of phagophore structures	[44]
Connexin 43	WD40 repeat domain	Inhibits autophagosome formation	[45,46]
TMEM59	WD40 repeat domain	Xenophagy	[47,48]
NOD1	WD40 repeat domain	Xenophagy	[49,50]
NOD2	WD40 repeat domain	Xenophagy	[47,50,51]
T3JAM	WD40 repeat domain	ND	[47]
DEDD2	WD40 repeat domain	ND	[47]
Ubiquitin	WD40 repeat domain	Xenophagy	[49,52]
PCM1	ND	Antigen presentation	[53]
Rab33A	CCD	Hormone secretion	[38,54]
TRIM20	WD40 repeat domain	Autophagic degradation of inflammasome components	[55]
PSMD1	N-terminal half; WD40 repeat domain	Autophagic degradation of PSMD1	[56]
PSMD2	WD40 repeat domain	Autophagic degradation of PSMD2	[56]

The proteins were listed according the order of appearance in this paper. ND: not determined.
